# Are Brief Alcohol Interventions Adequately Embedded in UK Primary Care? A Qualitative Study Utilising Normalisation Process Theory

**DOI:** 10.3390/ijerph14040350

**Published:** 2017-03-28

**Authors:** Amy O’Donnell, Eileen Kaner

**Affiliations:** Institute of Health and Society, Newcastle University, Newcastle upon Tyne NE2 4AX, UK; eileen.kaner@newcastle.ac.uk

**Keywords:** normalisation process theory, qualitative research, alcohol interventions, primary care

## Abstract

Despite substantial evidence for their effectiveness, the adoption of alcohol screening and brief interventions (ASBI) in routine primary care remains inconsistent. Financial incentive schemes were introduced in England between 2008 and 2015 to encourage their delivery. We used Normalisation Process Theory-informed interviews to understand the barriers and facilitators experienced by 14 general practitioners (GPs) as they implemented ASBI during this period. We found multiple factors shaped provision. GPs were broadly cognisant and supportive of preventative alcohol interventions (coherence) but this did not necessarily translate into personal investment in their delivery (cognitive participation). This lack of investment shaped how GPs operationalised such “work” in day-to-day practice (collective action), with ASBI mostly delegated to nurses, and GPs reverting to “business as usual” in their management and treatment of problem drinking (reflexive monitoring). We conclude there has been limited progress towards the goal of an effectively embedded preventative alcohol care pathway in English primary care. Future policy should consider screening strategies that prioritise patients with conditions with a recognised link with excessive alcohol consumption, and which promote more efficient identification of the most problematic drinkers. Improved GP training to build skills and awareness of evidence-based ASBI tools could also help embed best practice over time.

## 1. Introduction

Despite the substantial health, social and economic gains that could be realised through the effective implementation of screening (using a validated self-report questionnaire to identify problem drinkers [1]) and brief alcohol interventions (brief advice or behaviour change counselling to reduce problem drinking [2]), their organisation and provision in routine primary care remains inconsistent [3]. As a result, few eligible patients benefit from these simple, clinically- and cost-effective secondary prevention measures [4]. In England alone, Purshouse et al. estimate that screening all newly registered patients for excessive alcohol consumption, and delivering brief behavioural interventions to those identified at risk, would deliver 32,000 quality-adjusted life years (QALYs) at £6900 per QALY gained over a thirty year period [5].

Lack of training or suitable intervention materials [6,7], inadequate financial incentives [8,9], unsupportive specialist alcohol service provision [10], attitudinal factors [11,12] and everyday time pressures [13], have all been identified by general practitioners (GPs) and other health workers as barriers to the delivery of brief alcohol interventions [14,15]. Previous implementation research has tended to focus on testing strategies to improve health practitioners’ delivery of preventative alcohol care, such as via specific professional education and training, the use of financial incentives, or facilitated access to electronic or online screening and brief alcohol interventions [16,17,18]. Policy measures to encourage the delivery of brief alcohol interventions in routine primary care and other settings have included the development and dissemination of expert guidelines [19,20] and the introduction of targeted pay-for-performance schemes [21]. For example, the Scottish Government established a health improvement target (HEAT H4), supported by substantial investment, requiring the Scottish NHS to deliver 149,499 brief interventions across relevant settings, with mixed though broadly positive effects [22]. Similar initiatives were introduced in Sweden [23] and Finland [24], also resulting in increased rates of intervention delivery, particularly in primary care. More recently, in England, alcohol consumption questions were incorporated within NHS Health Check for adults aged 40–75 [25], and in April 2015, financial incentives for alcohol were replaced by a contractual requirement for practices to identify newly registered adult patients drinking above recommended levels [26].

However, despite such initiatives, we are little further forward in understanding “what works” when it comes to encouraging primary care clinicians to consistently deliver brief alcohol interventions in routine practice. Small to moderately sized effects demonstrated in previous research rarely persist over time. Moreover, outside the relative artifice of a research study setting, there is limited published evidence on the extent to which such strategies might be impactful in the “real world”, although the use of financial incentives to stimulate activity shows some promise [27,28,29].

Implementation theory can help illuminate our understanding of how evidence translates into real world practice in complex health care systems [30]. Normalisation Process Theory (NPT) provides a robust theoretical framework to help us consider how healthcare practices are: organised and operationalised (their implementation); routinized in everyday practice (their embedding); and sustained within the wider social context (their integration) [31]. NPT proposes four core constructs that represent the different kinds of “work” that people do when implementing a new practice (technology, or intervention): *coherence; cognitive participation; collective action; and reflexive monitoring* (see Figure 1) [32].

We used NPT to help understand the barriers and facilitators experienced by English GPs as they implemented alcohol prevention activities in routine clinical practice.

The interviews were conducted as part of a mixed-methods investigation of the impact of financial incentives on alcohol brief intervention delivery in Northern England. Under the voluntary national alcohol-related risk reduction Directed Enhanced Service (DES) scheme, participating practices were paid £2.38 for each newly registered adult patient recorded as being screened for heavy drinking using a validated self-report screening questionnaire [25]. Whilst the scheme did not directly remunerate intervention delivery, contractual guidance made clear that those patients identified as risky drinkers should receive an appropriate intervention based on the “How Much Is Too Much?” programme and related suite of materials [33]. Locally-negotiated Enhanced Service (LES) schemes for alcohol were also introduced in certain areas. LES schemes varied in their scope and reimbursement rates, although generally they were more generous than their national counterparts, and involved a more opportunistic approach to screening. In this area of Northern England, practices received £8.00 for each registered patient aged 16+ (excluding newly registered patients covered by the DES), who screened positive for risky drinking and received brief alcohol advice. In addition to the screening and intervention approach outlined in the DES guidance, the LES also required all clinicians involved in delivering the scheme to have undertaken appropriate training, such as SIPS for Level 1 [34].

The study comprised two components. First, the impact of financial incentives on screening and brief alcohol interventions were assessed by comparing recorded rates of delivery between those practices receiving financial incentives for alcohol screening and those not receiving additional payments [29]. Second, semi-structured interviews were used to explore GPs’ perceptions of factors influencing the delivery and recording of alcohol interventions in routine consultations in their practices. GPs were selected as interview subjects as opposed to other practice staff such as nurses, in recognition of the pivotal role they perform in directing, delegating and delivering alcohol prevention activities in English primary care [35,36]. In this paper, we report an in-depth NPT-informed qualitative analysis of the views of GPs on the implementation of screening and brief alcohol interventions in routine primary care.

## 2. Materials and Methods

In-depth semi-structured interviews were used to explore factors influencing GPs’ delivery of screening and brief alcohol interventions in day-to-day practice. Ethical approval for the study was obtained from Newcastle and North Tyneside Research Ethics Committee 1 (10/HO906/47) and all participants gave written informed consent to be interviewed.

### 2.1. Setting and Participants

Interviewees were recruited from GP practices based across two NHS organisational areas in Northern England, encompassing six former primary care trusts. In Area A, there had been limited take-up of the national DES scheme for alcohol. In Area B, universal coverage of the national scheme had been achieved across practices, as well as an additional LES scheme introduced by the respective health authority. A purposeful sampling strategy was employed to identify a wide diversity of participant perspectives. Alongside the focus criteria of interest to the study, NHS area and incentive status, we also sought to achieve a variation in terms of participants’ gender, experience in practice and employment status (salaried or practice partner), as previous literature suggested these factors were also of relevance [37]. Practice managers were asked to nominate potential interview participants, and GPs were then contacted via telephone or email by AOD, at which point the confidential and anonymous nature of the study was emphasized, and written informed consent obtained.

### 2.2. Data Collection

Interview data were collected between March 2012 and May 2013. Interviews took place at a time and location convenient to the participant, generally at their practice, and were conducted by AOD. The mean length of the interviews was 32 min (range: 22–47). Questions sought to elicit GPs’ perspectives on the process of delivering screening and brief alcohol interventions, focusing on the feasibility, acceptability and effectiveness of tackling risky drinking in their practices, including the involvement of other team members, during routine consultations with patients. In particular, practice-level approaches to case identification were examined, such as targeted versus universal screening approaches, and the use of validated screening questionnaires as opposed to alternative and/or more informal methods. A topic guide was used to focus discussions around these broad questions of interest (see Figure 2), however emergent issues were also pursued as appropriate. Additionally, to stimulate a reflective approach to the interview process, and to further inform analysis, fieldwork diaries were kept. Interviews were conducted until data saturation occurred.

### 2.3. Data Management and Analyses

Interviews were audio-recorded, transcribed verbatim, checked and anonymised. Data analysis took place in two stages. First, we used Framework Analysis to sift, chart and sort the interview data in accordance with key issues and themes in a five step process: (1) familiarization (reading and re-reading the transcripts alongside contextual data gathered via fieldwork diaries); (2) identifying a thematic framework (based on line-by-line deductive and inductive coding of an initial sample of transcripts, primarily themed around the topic guide but also incorporating any emergent issues of interest); (3) indexing (application of the working thematic framework to the remaining transcripts); (4) charting (summarising data from each transcript against the framework); and (5) mapping and interpretation (exploring characteristics of, and differences/relationships between the data through discussion) [38]. AOD conducted the initial analyses, with emergent themes discussed at data analysis meetings with EK, and refined or further developed as necessary. In stage two, we mapped emergent data themes against the four main constructs of NPT, checking for fit, and exploring areas of divergence and discrepancy. This two stage approach, used in previous similar studies, helped to avoid “forcin” our data into predetermined conceptual categories and thus ensured our interpretation remained data-driven [39]. NVivo Qualitative Research software (v11: QSR International, Cambridge, MA, USA) was employed to support data management and analysis.

## 3. Results

In total, 14 GPs were interviewed, with an equal representation of practices from each NHS area, and a broad balance of gender, experience, employment and incentive status. Full participant details are provided in Table 1.

The findings are structured around the four main constructs of NPT (coherence, cognitive participation, collective action, reflexive monitoring) with illustrative quotations from the GP interviews. Table 2 illustrates how the emerging themes and sub-themes map onto these key constructs. All initial narrative themes from stage one of analysis were successfully mapped against the first three constructs (coherence, cognitive participation, and collective action). However, by applying a NPT-driven approach to analysis in stage two, we also identified an additional sub-theme under “reflexive monitoring”, which concerned the tendency for GPs to revert to “business as usual” in their approach to screening and brief alcohol intervention delivery over time. Data are presented to support the analysis, labelled by participant identifier (GP = general practitioner), gender and practice incentive status.

### 3.1. Coherence—Making Sense of Screening and Brief Interventions for Alcohol

The extent to which GPs “make sense” of the practices and assumptions that comprise screening and brief alcohol interventions is fundamentally important in ensuring their effective delivery in routine care. Whilst only a small number of participants had previously experienced any formal training, the interviews suggested that most GPs were familiar with screening and brief alcohol interventions, and demonstrated a reasonable understanding of their purpose. In terms of the value and usefulness of available screening tools such as the AUDIT questionnaire and its shorter versions, several GPs expressed an appreciation of the structure and formality they helped provide. Their usefulness was often expressed in terms of offering some sort of aide memoire that GPs could refer back to as needed to prompt them to ask the key screening questions. For example, one GP commented that:

“*By and large yes they are, they’re helpful… they certainly remind you of which questions to ask and perhaps fill in some of the details that you sometimes don’t ask*.”

**GP2, male, directed enhanced service**

However, GPs were not always able to discriminate between using a structured questionnaire to assess alcohol-related harm or risk as opposed to just asking a simple consumption question (i.e., how many units of alcohol do you drink per week?). Most either expressed a preference for using consumption questions or viewed them as eliciting the equivalent information from a patient. In addition, interviewees struggled to distinguish between the separate, albeit interrelated, components of the screening and intervention process. That is, the lines between first screening a patient, and second delivering advice on the basis of the screening score, were often blurred into one somewhat amorphous process. The following quote is fairly illustrative of the overall confusion in this respect:

“… *where there are issues that alcohol comes up, then you would possibly use the brief intervention screening. Probably not as often as we should I would imagine. We probably ask more about alcohol and do our own sort of version of brief interventions rather than use the formal screening tool*.”

**GP2, male, directed enhanced service**

### 3.2. Cognitive Participation—Investing in Preventative Care for Risky Drinking

Making sense of the intervention and related practices is only one type of “work” that GPs need to perform if screening and brief alcohol interventions are to be adequately embedded: they must also engage with the intervention, on both an individual as well as a practice level. In this respect, whilst GPs appeared relatively comfortable with the purpose and process of screening and brief alcohol interventions, such understanding did not necessarily translate into a willingness to play an active role in their implementation.

This dissonance between *understanding* and *investing*, appeared to stem from GPs’ mixed views on “role legitimacy” (perceived boundaries of professional responsibility and the right to intervene) in relation to alcohol prevention work, and in particular, around the screening process. Interviewees described themselves as primarily focused on delivering the “patient-centered consultation”: as one interviewee put it *“a good GP will always stay on the patient’s agenda”* (**GP12, female, local and directed enhanced service**). There was a perceived conflict between delivering personalised, patient-centred care and following the pre-determined script of an alcohol screening questionnaire. Another GP commented:

“… *tying someone down to the number of units per week can take quite a long time and can disrupt the flow of the consultation…for my purposes, if the patient will or won’t acknowledge that it’s a problem is more important to me than specific numbers*…”

**GP11, male, local and directed enhanced service**

As such, many participants felt it was more legitimate for a GP to introduce the topic of alcohol if justifiably linked to a patient’s main presenting condition. For example:

“…*if somebody says it was insomnia and it transpired they were sleeping badly because they were drinking 70 or 80 units of alcohol a week then you would discuss it…If someone comes in with gout or something that would be a wonderful opener…but…we don’t proactively ask alcohol. We don’t go out to seek alcohol on its own*.”

**GP12, female, local and directed enhanced service**

In contrast, many interviewees appeared to feel that it was more appropriate, and indeed more realistic, for nurses to provide the majority of tasks involved in delivering screening and brief alcohol interventions in primary care. Two key factors contributed to this viewpoint. First, there was a perception that the structured approach of administering an alcohol screening questionnaire was more clearly aligned to the role, responsibilities and existing skillset of nursing staff. The following example demonstrates this perspective well:

“…*the practice nurses probably have a much more structured approach…most of their work is chronic disease management and they tend to be filling in screening tools. So that’s what they do all the time, so they’re much better at it*.”

**GP2, male, directed enhanced service**

Second, there was also an implication that because GPs needed to prioritise the main presenting condition, there was limited opportunity to raise additional concerns within the standard ten minute consultation slot. There was a perception that nurses had more time to address broader practice agendas, including preventative healthcare issues:

“…*the biggest challenge is time. Because almost entirely that’s not what the patient has come about you know, erm, if they come asking to speak about their alcohol I don’t think it’s, well it may happen a once a year… but the rest of the time they’ve come about something else and, and so to give that something else its full appointment’s worth and then also fit in a little bit of our own agenda that’s the most challenging thing*.”

**GP14, male, directed enhanced service**

### 3.3. Collective Action—Implementing Brief Alcohol Interventions

This concern to tailor the content of a consultation to the needs, preferences and capacities of the individual patient also shaped the way in which screening and brief alcohol interventions were operationalized by GPs in routine care. Several participants referred to the stages of behaviour change model as influencing their approach in this respect. For example one GP described how their delivery of a brief alcohol intervention would begin with an initial assessment of where an individual patient “sat” on the behavior change cycle.

“*You know, are they in a place where they’re actually thinking about it, or is it not even on their radar, or is it just, you know that’s what they’ve come to talk about? So that’s what I would do next*”.

**GP6, male, no enhanced service**

Other GPs mentioned drawing on available guidance such as the “How Much is Too Much?” programme [40], referring patients to online resources like the Drinkaware website (www.drinkaware.co.uk), and highlighting the short and long term impacts of excessive alcohol consumption. However, as well as influencing *how* GPs delivered alcohol interventions, the concept of stages of change also influenced *whether* interventions were delivered in the first place. For example, there was a belief that a patient needed to be “ready to change” for an alcohol intervention to be effective. As such, some GPs rationed their delivery of alcohol interventions to “changeable” patients, with this assessment of whether a patient was “ready to change” often being based on an instinctive “gut” reaction (*“sometimes…when you’re dealing with an individual in front of you, you get that kind of gut feeling that it is worth spending a bit of time”* (**GP8, male, local and directed enhanced service**)). Several interview accounts illustrated this perspective, for example:

“*I suppose one of the key things I feel with alcohol to some extent is, I suppose people have to be wanting to change before you can take them too far down the road of an intervention. And so sometimes yes, they know they’re drinking too much but they’re not that ready to change, so going through a whole pathway doesn’t always help*.”

**GP2, male, directed enhanced service**

Indeed, there were varied degrees of confidence expressed by interview participants in the actual effectiveness of brief alcohol interventions. Whilst most GPs believed that alcohol interventions could be effective in certain contexts, and with certain patients, there were also situations in which such an approach was viewed as unlikely to be impactful. For example:

“…*obviously there is evidence of brief interventions…but there is a perception that when it comes to lifestyle and things like that, people will just do what they want to do*.”

**GP8, male, local and directed enhanced service**

Further, and reflecting the views on role legitimacy expressed in the previous section, those participants based in practices that had signed up to voluntary financial incentives for alcohol suggested that the majority of incentivised activity (screening of newly registered patients) was either conducted by nurses or even indirectly by mail. Some implied that this resulted in a “tick-box” approach to screening:

“...*we tackle the DES through our new patient questionnaire that we post out to patients and they send it back and that fulfils the DES, you know I think it’s just a paper exercise*…”

**GP14, male, directed enhanced service**

Outside new patient registrations, screening activity also appeared to be embedded within annual patient health checks conducted as part of the management of long term conditions. However, once again, this meant that nurses or healthcare assistants were primarily responsible for their delivery. Talking about how the screening process was operationalised within their respective practice, one GP reported:

“…*that tends not to be done by the doctors, the AUDIT-C, that tends to be done by our healthcare assistants and nurses who are delivering the health promotion stuff, so everybody who comes through the hypertension clinic, the diabetic clinic, COPD, the asthma, the just the standard man off the street just wanting his cholesterol done, they all get fed through that template*.”

**GP12, female, local and directed enhanced service**

Another participant agreed:

“*We are encouraged to do opportunistic but I don’t think that happens, the way it tends to be done is as part of health checks so it’s more in people who have chronic diseases, so if they are seen about asthma, chronic heart disease any of those things*.”

**GP13, female, local and directed enhanced service**

### 3.4. Reflexive Monitoring—Evaluating and Modifying Brief Alcohol Interventions, and Embedding Change

Reflecting on the extent to which recent policy measures including financial incentives had changed GP practice around screening and brief alcohol interventions, most interviewees felt there had been limited impact. As one GP commented:

“*So while we’re signed up to it, and I think we are probably certainly asking people questions about their alcohol consumption at the relevant opportunities, such as new patient medicals and that sort of thing, we’re probably not doing anything too different from what we’d be doing anyway, which is just kind of dealing with stuff as it happens*.”

**GP8, male, local and directed enhanced service**

Another interviewee, who had previously participated in alcohol intervention training reported having since adapted (or deformalized) their approach:

“*I have been on the brief interventions training course although that now seems quite a long time ago. But we have, I suppose we tend to, I would admit that we probably don’t, we certainly don’t routinely screen every consultation or anything. So where there are issues that alcohol comes up, then you possibly use the brief intervention screening. Probably not as often as we should I would imagine. We probably ask more about alcohol and do our own sort of version of brief interventions rather than use the formal screening tool. Or I probably do but that’s because I’m not good at using screening tools*.”

**GP2, male, directed enhanced service**

A number of reasons appeared to influence this trend of GPs’ reverting to “business as usual” when it came to screening and brief interventions. For some, it was simply down to familiarity or habit. So, for example, several participants mentioned that they continued to use the CAGE screening tool, despite the fact they openly acknowledged its limitations in comparison to the “gold standard” of the AUDIT. For others, there was a perception that there was no longer a need to observe screening and brief alcohol interventions as an explicit process as they had absorbed the key components into their everyday routine (*“…I don’t follow a rigorous research script…I hope it’s integrated into my practice.”* (**GP7, male, local and directed enhanced service**)). Finally, and reflecting a common theme throughout the interviews, the failure of getting new intervention practices to “stick” stemmed from the ongoing challenge of integrating preventative alcohol work within the patient-centred consultation. As one GP commented:

“*I think it’s partly just because that’s the way we’ve always done things. That it’s just to do things from experience rather than reverting to tools. And I think partly because people usually consult with other problems and alcohol is a bi-product of the consultation. So often the screening is quite an add-on at the end and the screening tools are a bit more formal*.”

**GP2, male, directed enhanced service**

## 4. Discussion

This paper uses Normalisation Process Theory (NPT) as a framework to better understand barriers experienced by English GPs as they implement screening and brief alcohol interventions in routine practice. We identified multiple factors as detracting from this policy goal, which can be usefully mapped on to NPT constructs. In essence, although GPs were broadly cognisant and supportive of the principle of preventative alcohol care (coherence) this did not necessarily translate into a sense of personal investment in actual delivery of such interventions (cognitive participation). In turn, this lack of engagement shaped how GPs operationalised this “work” in day-to-day practice (collective action), with the delivery of screening and brief alcohol intervention mostly delegated to nurses or healthcare assistants, and GPs reverting to “business as usual” in their management and treatment of problem drinking (reflexive monitoring). “Business as usual” meant waiting for patients to raise the topic, or using more familiar and/or often less formalised approaches to asking about alcohol use than the validated screening tools prescribed in incentive frameworks. Moreover, this then followed through into employing an unsystematic and adaptive approach to intervention delivery. Practice was also influenced by GPs’ traditional perceptions of role legitimacy in relation to alcohol prevention work, underpinned by an overriding concern to deliver patient-centred care, where the patient’s immediate and individual needs are prioritised [41].

The main strength of this study is our use of NPT as an analytical lens through which to explore influences on GPs’ alcohol prevention practices. Various theories have been developed to support our better understanding of this complex process of adoption, integration and embedding of health interventions. These have included theories focussed on understanding the behaviour of the individual health professional, such as psychological theories of intention, and the Theory of Planned Behaviour [42]. However, it has been argued that such an essentially individualistic approach to implementation fails to take into account the multifarious and interrelated social and structural factors that might promote or constrain individual expressions of agency [31,43,44,45,46,47,48,49,50]. By drawing on this robust middle-range theory of implementation, we sought to take our understanding of the factors shaping the delivery of screening and brief alcohol interventions beyond those focussed on the behaviour of the individual health professional [51] to take into account the contextual factors that shape GPs’ practice over time [31,44,52]. Further, by focusing on GPs’ day-to-day or routine practices, our study provides insight into the challenges experienced by health care providers when they seek to translate research findings and policy directives into the real world. In particular, the introduction of local and national financial incentives to support implementation of screening and brief alcohol interventions in English primary care made for an especially interesting study context. Variability in the adoption of these incentives across our sample provided us with a natural experiment in which we could explore GPs’ views on the impact of external financial drivers on their practice [53].

One potential limitation is whether 14 GP interviews were sufficient to gain an adequately nuanced understanding of the complex processes and phenomena under investigation. Reflecting the challenges other studies have experienced in engaging GP participants in research [54], the rate of recruitment was indeed slow, and it was difficult to recruit participants from practices not incentivised for the delivery of alcohol interventions. At the same time, it is important to emphasize that we were recruiting only one type of practitioner, a relatively homogenous professional group, which shared critical similarities related to the research question [55]. Thus, despite the relatively small number of participants, data saturation was nevertheless judged to have been achieved, with a high level of consensus in viewpoints emerging from our analysis, despite efforts to probe and challenge during interviews [56]. Further and importantly, we managed to recruit from across the organisational areas of interest, which covered a range of alcohol enhanced service incentive systems, including those practices not incentivized for alcohol intervention delivery. In addition, our sample encompassed substantial variation in terms of the gender, employment status and experience of participants.

Some interviews were relatively brief, averaging at 32 min long overall. This reflected the time constraints of interviewing this busy group of health practitioners [54]. At the same time, it should be stressed that this was a professional, generally highly articulate group of interview participants, who in the main were able to quickly grasp and verbalise complex concepts, and willing to reflect on and question the issues discussed. Moreover, despite initial concerns that worries around “professional liability” could result in GPs providing more guarded, less truthful accounts of alcohol screening and brief intervention activity, particularly where such activities were being directly remunerated under either of the Enhanced Service schemes [57], this did not appear to be the case. Indeed, participants’ accounts were frequently candid, particularly on their (lack of) use of formal screening tools, and expressing widespread cynicism on the impact of financial incentives on service quality. It is also worth acknowledging that the GPs that took part in the interviews may be have been more interested in, and aware of, the topic of interest, and thus not necessarily representative of the wider GP community. As such, it is important to be mindful that the screening and brief alcohol intervention narratives described here may reflect more positive examples of this area of care.

Our study highlights the way in which competing demands on limited time can lead to the de-prioritisation of preventive care, and divergence from recommended clinical guidelines [7,58]. In particular, GPs were often using informal methods of assessing alcohol consumption in patients despite the fact that most were being specifically paid to use validated screening tools [59,60]. In contrast to other studies, we found little evidence to suggest that GPs find alcohol a particularly “sensitive” subject to discuss with their patients [7,11]. However, as has been found in other studies in this field, it was nevertheless clear that many GPs were unconvinced that patients would be receptive to advice about changing their drinking behaviour, particularly those patients drinking at heavy or dependent levels [7,61,62].

Interestingly, whilst previous research has identified lack of resources as a key barrier to screening and brief alcohol intervention delivery [8,9], with some positive examples of using financial incentives to stimulate activity (including studies by Hamilton et al. [28] and Khadjesari [63]), our findings paint a rather more complex picture. For GPs at least, it would appear that the use of pay-for-performance has failed to stimulate a significant change in practice around alcohol work. However, this was not necessarily the case at a practice level, where nurses and healthcare assistants were seen to be responsible for screening patients’ drinking behaviour, often in new patient registrations, and as part of the ongoing management of chronic conditions.

A key question is whether the piecemeal integration of screening and brief alcohol interventions in English primary care described here is problematic. If nurses are routinely screening patients for excessive alcohol consumption, and in turn providing advice to those in need of further support, then surely this is enough? Moreover, even if GPs seem reluctant to label their approach as a “brief alcohol intervention”, their descriptions of how problem drinking was managed in patient consultations suggested they may adhere to its basic principles, but that they modify practice to suit both their professional identity and real-world conditions. For some, rather than an “evidence to practice” gap, this rather represents a prime example of “normalisation”, whereby interventions are successfully moulded and integrated to meet the demands of local circumstances [64]. Thus maybe some “elasticity” is to be expected if clinicians are to negotiate the adoption of complex interventions within complex healthcare systems [65].

For others, however, the lack of benefit of screening and brief alcohol interventions reported in recent pragmatic trials in the field [66] serves to underline the risks involved in deviating too far from evidence-based practice [67]. Further, from the GP interview accounts in this study, whilst nurse-led screening may well be relatively routinized (at least for newly registered patients or those with long term conditions), there was little evidence to suggest that problem drinkers go on to receive appropriate support or advice. In part, this could reflect the design of the national DES for alcohol, which only directly incentivised the administration of a validated screening questionnaire. Thus one conclusion from this research would be that to effectively motivate practices to provide the full alcohol care pathway, governments need to reward intervention outcomes not just partial steps along the process. At the same time, accounts from GPs based in practices signed up to the local incentive scheme, in which delivery of brief advice was also incentivized, also suggested a piecemeal approach to provision of this element of care.

Evidence from a range of clinical fields highlights some risks and limitations of simply using financial incentives to stimulate change in professional practice [68]. Indeed, whilst incentives may have a positive impact on GPs’ engagement in delivering alcohol prevention work [69], previous research examining the challenge of translating screening and brief alcohol intervention to routine primary care nevertheless advocates a multi-stranded approach to implementation [16]. As such, maybe the more pertinent question is exactly which “strands” should such an approach comprise?

This study suggests three possible clinical levers that warrant future consideration.

- First, as GPs often prioritise treating the main presenting condition in their patients, one strategy would be to ensure that when there is a recognised link between problem drinking and particular health conditions, then screening and brief alcohol intervention is delivered. For example, there is good evidence for the impact of alcohol consumption, and especially heavy drinking, on raised blood pressure [70].
- Second, given the time pressured context of primary care, we may need to rethink screening approaches. Successfully identifying the one in four primary care patients likely to be drinking above recommended levels [18] could result in an untenable burden on health systems. The introduction of higher AUDIT cut-off levels could both ensure the most problematic drinkers are prioritised for support [4], at the same time as tackling resistance amongst busy practitioners who are concerned about the impact of false positives both on workload and their rapport with patients [12].
- Third, and finally, there is a need to ensure that the translation of screening and brief alcohol interventions into real world practice does not stray too far from the evidence-base [67]. There remains ongoing debate as to what exactly constitutes the “active ingredients” of brief alcohol interventions [71], and around the most cost- and clinically-effective screening strategy [67,72]. However, on the basis of the available evidence, clinicians should be reminded that validated screening questionnaires, even in their shortest forms, remain the most efficient tools to reliably identify problem drinkers, and that prompting self-recording of alcohol intake is associated with greater intervention effect sizes [73]. Training and support can help build GPs’ knowledge and skills in this area, and is associated with higher rates of intervention activity, which in turn boosts role security and GPs’ therapeutic commitment over time [69].

## 5. Conclusions

Our findings suggest that despite the introduction of a series of policy measures aimed at encouraging more screening and brief alcohol intervention delivery in primary care, the goal of an effectively embedded preventative care pathway for alcohol in English primary care remains elusive. Future policy should consider screening strategies that prioritise patients with conditions where there is a recognised link with excessive alcohol consumption, and which promote more efficient identification of the most problematic drinkers. Ongoing training and support for GPs to build skills and awareness of evidence-based screening and brief alcohol intervention tools can help embed best practice over time.

## Figures and Tables

**Figure 1:** Core constructs of Normalisation Process Theory.

**Table:** 

**Coherence:** is the “sense-making” work that people do individually and collectively when a new practice is implemented (e.g., to understand the differences between new and existing practice, to build a shared view of its purpose, to understand how it will affect them personally, and to grasp its potential benefits). **Cognitive participation:** is the “relational” work involved in engaging and legitimizing a new practice (e.g., whether individuals are prepared to invest in and/or sustain the intervention). **Collective action:** is the “operational” work performed by individuals or teams of professionals to organize and enact a new practice (e.g., the interactive work involved in reshaping relationships, policies and processes to accommodate the new practice, including the allocation of people and resources). **Reflexive monitoring:** is the “appraisal” work that people do to understand and evaluate the impact of a new practice (e.g., how it affects them and the people around them, and how individuals or groups come to decide whether the intervention is worthwhile or not over time).

**Figure 2:** Core questions and prompts used in GP interviews.

**Table:** 

**Q** Could you tell me about this practice? Prompts: Local area, size, history/recent notable changes, team composition, strengths/challenges. **Q** What is your role within this practice? Prompts: how long have you been based here; what are your particular interests/specialisms/responsibilities. **Q** How long have you been involved in delivering screening and brief alcohol interventions? **Q** Could you describe the process of delivering screening and brief alcohol interventions at this practice? Prompts: talk through process e.g., how do you identify potential problem drinkers; what happens next? **Q** How would you describe screening and brief alcohol interventions in terms of ease of delivery? Prompts: straightforward/challenging? Probe for specific examples. **Q** Do you think screening and brief alcohol interventions “work”? What evidence do you have? Prompts: own experience; patient outcomes; research evidence?

**Table 1:** Summary characteristics of general practitioners (GP) interview participants.

Sampling Criteria	Sub-Criteria	*N* (14)
**Gender**	Male	7
	Female	7
**Experience in practice**	>5 years	4
	5–15 years	3
	>15 years	7
**Employment status**	Partner	7
	Salaried GP	6
	Registrar	1
**Location**	Area A	7
	Area B	7
**Enhanced service status**	No Enhanced Service	3
	Directed Enhanced Service	4
	Directed Enhanced Service & Local Enhanced Service	7

**Table 2:** Summary of Normalisation Process Theory (NPT)-informed interview themes and sub-themes.

NPT Construct	Theme	Sub-Themes
Coherence	Making sense of screening and brief alcohol interventions	Validated screening tools interpreted as a useful framework to inform practice. Limited differentiation between individual components of screening and brief interventions.
Cognitive Participation	Investing in preventative interventions for alcohol	Perceived conflict between structured screening and alcohol interventions and patient-centred approach. Nurses viewed as better suited to delivering routine, task-based preventative care.
Collective Action	Implementing or day-to-day delivery of brief alcohol interventions	Patient-related factors influence GPs’ delivery of alcohol interventions. Financial incentives shape the organisation and provision of screening for problem drinking.
Reflexive Monitoring	Evaluating and modifying brief alcohol interventions, and embedding change	GPs revert to “business as usual” in their approach to preventative alcohol care over time.

## Abbreviations

AUDIT	Alcohol Use Disorders Identification Test
AUDIT C	Alcohol Use Disorders Identification Test Consumption
DES	Directed Enhanced Service
FAST	Fast Alcohol Screening Test
LES	Local Enhanced Service
NHS	National Health Service
